# Diffusion tensor imaging in frontostriatal tracts is associated with executive functioning in very preterm children at 9 years of age

**DOI:** 10.1007/s00247-020-04802-1

**Published:** 2020-09-01

**Authors:** Hanna Kallankari, Virva Saunavaara, Riitta Parkkola, Leena Haataja, Mikko Hallman, Tuula Kaukola

**Affiliations:** 1 PEDEGO Research Unit and Medical Research Center Oulu, University of Oulu, Oulu, Finland; 2 Department of Child Neurology, Oulu University Hospital, P.O. Box 23, FIN-90029 OYS, Oulu, Finland; 3 PET Center, Turku University Hospital, Turku, Finland; 4 Department of Medical Physics, Turku University Hospital, Turku, Finland; 5 Department of Radiology, University of Turku and Turku University Hospital, Turku, Finland; 6 Department of Child Neurology, Children and Adolescents, University of Helsinki and Helsinki University Hospital, Helsinki, Finland; 7 Department of Neonatology, Oulu University Hospital, Oulu, Finland

**Keywords:** Children, Diffusion tensor imaging, Frontostriatal tracts, Magnetic resonance imaging, Premature birth, Tractography, White matter

## Abstract

**Background:**

Very preterm birth can disturb brain maturation and subject these high-risk children to neurocognitive difficulties later.

**Objective:**

The aim of the study was to evaluate the impact of prematurity on microstructure of frontostriatal tracts in children with no severe neurologic impairment, and to study whether the diffusion tensor imaging metrics of frontostriatal tracts correlate to executive functioning.

**Materials and methods:**

The prospective cohort study comprised 54 very preterm children (mean gestational age 28.8 weeks) and 20 age- and gender-matched term children. None of the children had severe neurologic impairment. The children underwent diffusion tensor imaging and neuropsychological assessments at a mean age of 9 years. We measured quantitative diffusion tensor imaging metrics of frontostriatal tracts using probabilistic tractography. We also administered five subtests from the Developmental Neuropsychological Assessment, Second Edition, to evaluate executive functioning.

**Results:**

Very preterm children had significantly higher fractional anisotropy and axial diffusivity values (*P<*0.05, corrected for multiple comparison) in dorsolateral prefrontal caudate and ventrolateral prefrontal caudate tracts as compared to term-born children. We found negative correlations between the diffusion tensor imaging metrics of frontostriatal tracts and inhibition functions (*P<*0.05, corrected for multiple comparison) in very preterm children.

**Conclusion:**

Prematurity has a long-term effect on frontostriatal white matter microstructure that might contribute to difficulties in executive functioning.

## Introduction

The survival of infants born before 32 weeks of gestation has increased remarkably in the last 20 years. Neurologic outcomes have improved, as well, including a decreasing rate of major neurosensory disabilities such as cerebral palsy [1]. Despite this, children born at a very low gestational age (VLGA) continue to have difficulties in neurocognitive processing through their early school years and into adulthood [2–4], even with no serious perinatal brain injury [5]. Specific neurodevelopmental deficits, including problems in executive functioning, might explain poorer academic performance in this high-risk population [6].

Executive functions comprise cognitive processes such as attentional control, inhibition, shifting, working memory, reasoning and problem-solving. The prefrontal cortex and frontostriatal tracts are part of a wide neural network that conveys these processes [7, 8]. Among cortical areas, the frontal cortex has the longest maturational time window [9], making it particularly susceptible to both structural and functional alterations.

Very preterm birth might affect myelination and axonal growth in developing white matter. Diffusion tensor imaging (DTI) is a special sequence of MRI used to study white matter microstructure [10]. Fractional anisotropy, axial diffusivity, radial diffusivity and mean diffusivity are quantitative measures of water diffusion. These DTI metrics offer the opportunity to study possible correlations between voxel-based changes in white matter microstructure and neurodevelopmental outcomes in children born very preterm [11].

As part of our follow-up study, a cohort of VLGA children and a comparison group of term children underwent DTI and neuropsychological assessments at 9 years of age. Previously, we showed that VLGA children at school age had more problems in executive functions when compared to term children [3]. In the present study, we defined frontostriatal tracts using probabilistic tractography and quantitated DTI metrics. Our aim was to test the hypotheses that the DTI metrics of frontostriatal tracts differ between VLGA and term-born schoolchildren and that these microstructural properties correlate to executive functioning among schoolchildren born very preterm.

## Materials and methods

### Subjects

The present study was part of a prospective follow-up cohort study of children born before 32 weeks of gestation at Oulu University Hospital between November 1998 and November 2002. The data set of this population has been described in detail [3, 12]. The VLGA children underwent serial brain US examinations during the neonatal period, and severe brain injury was identified as intraventricular haemorrhage Grades 3 or 4 [13] or cystic periventricular leukomalacia [14]. A group of age- and gender-matched term children was selected from the birth register of Oulu University Hospital at the age of 9. The recruitment and inclusive follow-up assessments were carried out during a 4-year period, between November 2007 and November 2011, at Oulu University Hospital [3, 12].

Altogether, 68 VLGA children and 23 term children underwent brain MRI at a mean age of 9 years (range 8.6–9.6 years). Fourteen VLGA children were excluded from the current study. The exclusion criteria were cerebral palsy (*n*=4), missing DTI data (*n*=1), problems in MRI data transfer (*n*=2) or with quality control criteria (*n*=5), and technical problems in fibre tracking (*n*=2). Three term children were excluded: two because of missing DTI data and one because of technical problems in fibre tracking. Five parents of VLGA children refused to participate in neuropsychological tests, leaving 49 VLGA children and 20 term children with results from neuropsychological assessments at a mean age of 9 years (range 8.7–9.3 years). The ethics committee of Oulu University Hospital approved the study (reference number 60/2007), and we obtained written informed consent from both the participating children and their parents.

### Neurologic and neuropsychological assessments

Severe neurologic impairment was defined as cerebral palsy. Among the VLGA children, cerebral palsy was confirmed by a child neurologist at Oulu University Hospital at the age of 5 years. The diagnosis was based on standard criteria by the Surveillance of Cerebral Palsy in Europe network [15]. Every child participating in the current study also underwent a structured neurologic assessment at the age of 9 years. None of the term children was diagnosed to have cerebral palsy. All participants attended mainstream school.

Neuropsychological assessments were performed by a child psychologist at Oulu University Hospital. The children completed 14 subtests from the Developmental Neuropsychological Assessment, Second Edition [16]. Standardised scores were calculated and analysed for these subtests. The subtest scores, with a range of 1–19, had a normed mean of 10 and a standard deviation of 3. The five subtests describing executive functioning —auditory attention, response set, inhibition/naming, inhibition/inhibition and inhibition/switching — were chosen for the present study from the Developmental Neuropsychological Assessment, Second Edition. To reduce the number of outcome variables, we further calculated mean scores for attention domain (comprising auditory attention and response set subtests) and for inhibition domain (comprising three inhibition subtests). Four VLGA children and one term child could not perform the inhibition/switching subtest, leaving 45 VLGA children and 19 term children with no severe neurologic impairment and with results from the inhibition domain.

### Neuroimaging

Conventional MRI was performed using a 1.5-tesla (T) GE Signa HDX (GE Healthcare, Milwaukee, WI). The study protocol comprised a T1-weighted sagittal spin-echo sequence. For this protocol, the slice thickness was 5 mm with a 1-mm gap between slices, the field of view was 24 cm with a 512×512 matrix, and the repetition time (TR)/echo time (TE) was 540/14 ms. In addition, T2-weighted axial images were taken using the Propeller technique with a slice thickness of 5 mm and a 1-mm gap, an echo train length of 28, a reconstruction diameter of 22 cm with a 512×512 matrix, and a TR/TE of 5,000/173 ms.

DTI was obtained using a spin-echo echoplanar sequence with an isotropic 3-mm voxel, 40 directions and a b value of 1,000. The repetition time was 9,000 ms, and the echo time was as short as possible. The slice thickness was 3 mm, and the field of view was 19.2 cm with a 64×64 matrix.

We used an 8-channel head coil. The child’s head was surrounded by soft cushions during scanning, and ear plugs were used to protect the child from imaging noise. No sedation was used during imaging. Data quality control was carried out with DTIPrep (Universities of North Carolina, Iowa and Utah, USA) [17]. Data were checked for slice-wise and interlace-wise intensity differences. Eddy current and motion defects were corrected, and data were checked gradient-wise for residual motion or deformations. Gradient directions that had image artefacts were removed from the data.

Probabilistic tractography was carried out using the Functional Magnetic Resonance Imaging of the Brain (FMRIB) Diffusion Toolbox version 3.0 (FMRIB Analysis Group, Oxford, UK), which allowed for an estimation of the most probable pathway location from a seed point using Bayesian techniques [18]. Fibre tracking was initiated from all voxels within the seed masks to generate 5,000 streamline samples, each with a step length of 0.5 mm and a curvature threshold of 0.2.

**Fig. 1:**
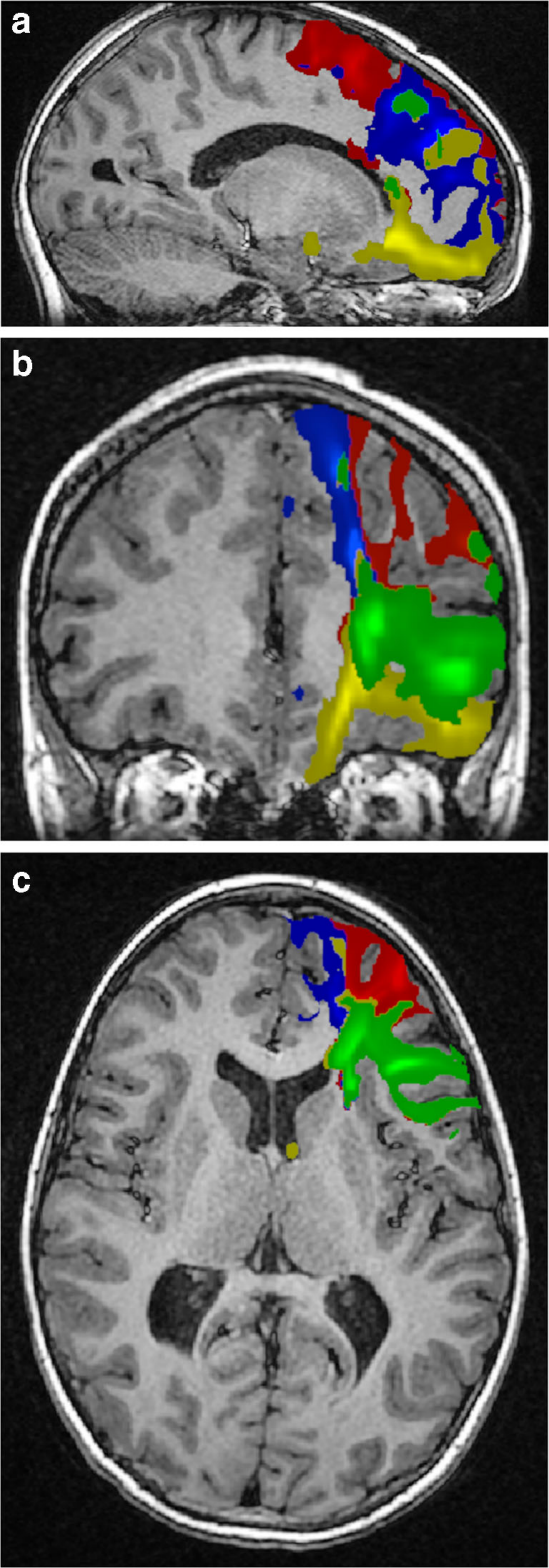
T1-weighted MR images with an overlay of the frontostriatal tracts coded by colours in a 9.1-year-old healthy boy (a control) who had normal findings at conventional MRI. **a** Sagittal. **b** Coronal. **c** Axial. The dorsolateral prefrontal caudate tract is red, the medial prefrontal caudate tract is blue, the orbitofrontal caudate tract is yellow and the ventrolateral prefrontal caudate tract is green

The frontostriatal fibres were divided into four bundles using a regions-of-interest approach. We used a connectivity-based seed classification analysis to identify connections among the dorsolateral prefrontal cortex, medial prefrontal cortex, orbitofrontal cortex, ventrolateral prefrontal cortex and caudate nucleus (Fig. 1). The cortex areas were used as seed masks. The caudate nucleus was used as a waypoint and a stop mask. Connections were analysed bilaterally. The contralateral hemisphere, ipsilateral putamen and thalamus were used as avoid masks. Masks were constructed using MARINA software (Bender Institute of Neuroimaging, University of Giessen, Germany) [19] and were originally defined in the Montréal Neurological Institute space and later transformed to diffusion space using FMRIB’s Linear Image Registration Tool with default settings.

Voxel values represented the number of samples passing through any given voxel. Connectivity distribution was normalised using the waytotal value. To remove voxels with very low connectivity, we applied a probability threshold using values selected from the normalised connectivity distribution. The robust intensity range was used to read the two percentile values from healthy controls. The mean of these values was calculated and used as a threshold of the normalised connectivity distribution. The threshold varied among the tracts. The created image was binarised and used as a mask to collect fractional anisotropy, mean diffusivity, axial diffusivity and radial diffusivity values from each child in the study cohort.

### Statistical analyses

Statistical analyses were performed using SPSS 24.0 (IBM, Armonk, NY). Differences in the averaged DTI values (fractional anisotropy, mean diffusivity, axial diffusivity and radial diffusivity) in each tract and in both sides between VLGA and term-born children were evaluated using the Student’s *t*-test. Linear regression analyses were conducted to adjust these results with gender and perinatal brain injury. Effect sizes were calculated in terms of Hedges’s g using means and standard deviations [20]. A commonly used interpretation is to refer to effect sizes as 0.20, 0.50 and 0.80 for small, medium and large, respectively. We evaluated differences in the composite scores of attention and inhibition domains between VLGA and term-born children using the Student’s *t*-test. Further, we performed linear regression analyses to evaluate correlations between executive function scores and DTI values among VLGA children while controlling for gestational age, gender and perinatal brain injury. We controlled for inflation of Type I error by reducing the number of comparisons; only DTI values and executive function scores that differed significantly between the VLGA and term children were included in these analyses. The results were controlled for multiple comparisons using the false discovery rate method developed by Benjamini and Hochberg [21]. The level of significance was set at *P<*0.05, two-tailed.

## Results

Antenatal and neonatal clinical characteristics of the study population are shown in Table 1. Four VLGA children had severe perinatal brain injury. The VLGA children were found to have significantly higher fractional anisotropy and axial diffusivity values in the left and right dorsolateral prefrontal caudate tracts and in the left and right ventrolateral prefrontal caudate tracts then the term children (Table 2). These results remained significant after controlling for gender and perinatal brain injury using linear regression analyses and after correcting for multiple comparisons. There were no significant differences in radial diffusivity and mean diffusivity values between the groups (data not shown). VLGA children had a 1.4-point (95% confidence interval [CI] 0.4–2.4; *P*=0.005) reduction in inhibition domain scores compared with term children. No significant differences were found in attention domain scores between the groups (*P*=0.341).

**Table 1:** Clinical characteristics of study populations

	VLGA children (*n=*54)	Term children (*n=*20)
Boys, *n* (%)	29 (54)	11 (55)
Singleton, *n* (%)	36 (67)	17 (85)
Gestational age, mean in weeks (range)	28.8 (24.4–31.9)	39.4 (37.3–41.6)
Birth weight, mean in grams (range)	1,169 (538–2,295)	3,356 (2,655–4,040)
Foetal growth restriction, *n* (%)	12 (22)	0
Antenatal steroids, *n* (%)	46 (85)	0
Intraventricular haemorrhage Grade 2, *n* (%)	2 (4)	0
Intraventricular haemorrhage Grade 3, *n* (%)	3 (6)^a^	0
Cystic periventricular leukomalacia, *n* (%)	1 (2)	0
Age at the DTI scanning, mean in years (range)	9.0 (8.6–9.7)	9.1 (8.8–9.3)

*DTI* diffusion tensor imaging, *VLGA* very low gestational age

^a^None of the VLGA children had intraventricular haemorrhage Grade 4

**Table 2:** Comparisons of fractional anisotropy and axial diffusivity values of the frontostriatal tracts in very low gestational age (VLGA) and term children

	Fractional anisotropy				Axial diffusivity (×10^−3^ mm^2^/s)			
Tract	VLGA^a^ (*n=*54)	Term^a^ (*n=*20)	*P*^b^	Hedges’s g^c^	VLGA^a^ (*n=*54)	Term^a^ (*n=*20)	*P*^b^	Hedges’s g^c^
DLPFC, left	0.260 (0.027)	0.243 (0.016)	0.001^d^	0.692	1.24 (0.047)	1.21 (0.029)	0.001^d^	0.698
DLPFC, right	0.253 (0.019)	0.240 (0.014)	0.008^d^	0.730	1.25 (0.046)	1.21 (0.041)	0.007^d^	0.894
MPC, left	0.260 (0.029)	0.260 (0.016)	0.533	0	1.24 (0.046)	1.23 (0.041)	0.236	0.224
MPC, right	0.273 (0.024)	0.270 (0.016)	0.588	0.135	1.24 (0.043)	1.24 (0.051)	0.824	0
OFC, left	0.244 (0.029)	0.238 (0.020)	0.419	0.222	1.20 (0.051)	1.19 (0.043)	0.666	0.204
OFC, right	0.239 (0.025)	0.229 (0.019)	0.098	0.424	1.17 (0.048)	1.17 (0.042)	0.976	0
VLPFC, left^e^	0.292 (0.030)	0.260 (0.018)	<0.001^d^	1.170	1.18 (0.057)	1.14 (0.031)	0.005^d^	0.778
VLPFC, right^e^	0.271 (0.022)	0.252 (0.017)	0.001^d^	0.914	1.16 (0.033)	1.14 (0.033)	0.010^d^	0.606

*DLPFC* dorsolateral prefrontal caudate, *MPC* medial prefrontal caudate, *OFC* orbitofrontal caudate, *VLPFC* ventrolateral prefrontal caudate

^a^Data are given as mean (standard deviation)

^b^Student’s *t*-test. *P*<0.05 is significant

^c^Effect sizes were calculated in terms of Hedges’s g

^d^The results remained significant after adjusting for gender and perinatal brain injury and after correcting for multiple comparisons

^e^The diffusion values of left and right VLPFC tracts were not obtained from three VLGA children because of technical problems in fibre tracking

Correlations between inhibition domain scores and DTI metrics (fractional anisotropy and axial diffusivity values) in the left and right dorsolateral and ventrolateral prefrontal caudate tracts among VLGA children were assessed using linear regression analyses. High fractional anisotropy values in these tracts correlated with low inhibition domain scores. In addition, high axial diffusivity values in the right dorsolateral prefrontal caudate tract correlated with low inhibition scores. The results remained significant after adjusting for gestational age, gender and perinatal brain injury and after correcting for multiple comparisons (Table 3).

**Table 3:** Correlations between inhibition scores and fractional anisotropy and axial diffusivity values of the dorsolateral prefrontal caudate (DLPFC) and ventrolateral prefrontal caudate (VLPFC) tracts among very low gestational age (VLGA) children with no severe neurologic impairments (*n=*45)

		Inhibition domain	
Tract	DTI value	r^a^	*P*^b^
DLPFC, left	Fractional anisotropy	−0.443	<0.001^c^
DLPFC, right	Fractional anisotropy	−0.449	<0.001^c^
VLPFC, left^d^	Fractional anisotropy	−0.404	0.003^c^
VLPFC, right^d^	Fractional anisotropy	−0.461	<0.001^c^
DLPFC, left	Axial diffusivity	−0.050	0.710
DLPFC, right	Axial diffusivity	−0.317	0.011^c^
VLPFC, left^d^	Axial diffusivity	−0.049	0.719
VLPFC, right^d^	Axial diffusivity	−0.220	0.106

*DTI* diffusion tensor imaging

^a^Regression coefficient

^b^Linear regression analysis adjusted for gender, gestational age and perinatal brain injury. *P*<0.05 is significant

^c^The results remained significant after correction for multiple comparisons

^d^The diffusion values of left and right VLPFC tracts were not obtained from two VLGA children because of technical problems in fibre tracking

## Discussion

Consistent with our hypothesis, this study showed microstructural differences in the frontostriatal tracts between VLGA children with no severe neurologic impairment and term children at 9 years of age. The VLGA children had significantly higher fractional anisotropy and axial diffusivity values in two frontostriatal tracts bilaterally — dorsolateral prefrontal caudate and ventrolateral prefrontal caudate — than did the term children. Further, fractional anisotropy and axial diffusivity values in these tracts correlated negatively with neuropsychological test scores measuring inhibition, one of the core executive functions, in the VLGA children.

Functional and structural neuroimaging studies have demonstrated that during its maturational process the prefrontal cortex (including the dorsolateral prefrontal, medial prefrontal, orbitofrontal and ventrolateral prefrontal) forms connections within itself and with other cortical and subcortical brain regions [7]. Despite this widespread neural circuitry, prefrontal cortex and its frontostriatal network are still thought to play a major role in guiding processes required for flexible and goal-directed behaviours [7, 8, 22]. Preterm birth is associated with both reorganization and disruptions in cortical–cortical and cortical–subcortical connectivity [23].

Commonly, fractional anisotropy values have been found to increase during white matter maturation, reflecting myelination, more organised axons, and thus restricted water diffusion [24], in most cases with preterm children having lower values compared to term-born controls [25]. The present study found higher fractional anisotropy and axial diffusivity values in two frontostriatal tracts bilaterally in VLGA children compared to term children. Supporting our findings, recent studies have also found higher fractional anisotropy values in certain white matter pathways among different age groups of individuals born preterm [26–30]. A meta-analysis, based on 13 studies and including participants from children to young adults born preterm, identified four regions of increased fractional anisotropy and 11 regions of decreased fractional anisotropy in preterm subjects compared to term controls [25]. In addition to our study, others have reported higher axial diffusivity values in certain white matter regions, including the reticular activating system involved in attention abilities [31], among preterm individuals compared to term-born controls [27, 29, 30].

One explanation for the variability of fractional anisotropy among studies might be the differences in imaging protocols and analytical methods among studies [5, 25]. On the other hand, both tracts in the current study found to have increased fractional anisotropy values run from the prefrontal cortex, known to have long maturational time window. Thus, although the MRI was performed in VLGA and term children at same chronological age, the biological maturation profile might differ between these two cohorts, and this difference might be reflected in DTI metrics in this brain area. It has also been suggested that high fractional anisotropy values relate to increased myelination as a marker of compensatory recovery process after early white matter damage [28]. However, other factors in white matter such as axonal status and crossing fibres in voxel level could also affect these microstructural properties [26, 28, 32].

Previously, we demonstrated that VLGA children scored worse in inhibition/naming, inhibition/inhibition and inhibition/switching compared to term children at 9 years of age [3]. The current study showed that high fractional anisotropy values in the frontostriatal tracts, bilateral dorsolateral prefrontal caudate and bilateral ventrolateral prefrontal caudate were associated with poor performance in inhibition tasks. In contrast to our findings, previous studies have demonstrated correlations between low fractional anisotropy values in different white matter areas and poor executive functioning among very preterm populations [5, 31, 33, 34]. In addition, both negative and positive correlations between executive functions and fractional anisotropy in the different segments of the corpus callosum were found in 6-year-old preterm children [35]. Other studies have found no significant correlation between fractional anisotropy and executive functions [36, 37]. However, the tests measuring executive functioning in previous studies were dissimilar, and white matter areas evaluated in the present and previous studies differed from one another. The correlation variability between DTI metrics and neurocognitive functions could also stem from differences in vulnerability or the developmental stages of specific tracts [38]. Previously, VLGA individuals with higher fractional anisotropy in certain corticospinal and corpus callosal tracts had worse outcome in fine motor and executive functions, respectively, indicating — in line with our study — that higher fractional anisotropy values do not always imply better function [35, 39]. While projecting from the frontal lobe to the caudate nucleus, the frontostriatal tracts cross with callosal and association fibres [8]. Thus, it is possible that the correlation between increased fractional anisotropy values in dorsolateral prefrontal caudate and ventrolateral prefrontal caudate tracts and unfavourable executive functioning might be related to the effect of crossing fibres or might reflect axonal loss and disrupted branching in those white matter areas [26, 28, 32].

Among the strengths of the present study, we consider that our VLGA population belonged to the well-defined prospective cohort [3, 12]. In addition, the study included a comparison group of term children. All the children underwent imaging and neuropsychological assessments within the same age range. The same MRI scanner was used to obtain MRI sequences for all participants. DTI is known to be prone to artefacts, particularly from motion. In the current study, data quality control was carried out with DTIPrep [17]. Data were checked for slice-wise and interlace-wise intensity differences. Eddy current and motion defects were corrected, and data were checked gradient-wise for residual motion or deformations. Gradient directions that had image artefacts were removed from the data. The DTI analyses were performed using well-defined techniques [18, 19]. The evaluation of executive functioning was based on an objective and standardised method [16]. The size of our VLGA group corresponded with the size of previous DTI study groups [25, 27, 30]. To avoid Type I error, multiplicity of the analyses was controlled, and correction for multiple comparison was used. A potential concern for our study was the small group size of term children, which could have affected hypothesis testing and could have resulted in Type II error. However, the group of term children was age- and gender-matched, randomly recruited from the Oulu University Hospital birth register, and comprised a representative sample of 9-year-old schoolchildren with no severe neurologic impairment.

## Conclusion

Very preterm children had higher fractional anisotropy and axial diffusivity values in diffusion tensor imaging bilaterally in two frontostriatal tracts when compared to term children at a comparable 9 years of age. Furthermore, the diffusion values of frontostriatal tracts correlated negatively with neuropsychological measures of inhibition in the very preterm children. These results indicate that white matter microstructure is different in very preterm children — even with no severe neurologic impairment — when compared to term children and that this difference might reflect the complex maturational processes of specific neurodevelopmental abilities at school age.
